# A Case in Practice

**Published:** 1882-03

**Authors:** J. B. Tullis


### A CASE IN PRACTICE.

BY J. B TULLIS.

In the spring of 1876 I was sent for to see a lady, living four and one-half miles in the country, with a request to bring my instruments to take out all her teeth. The previous night she had suffered much, and her physician had given her morphia several times to relieve pain and give rest, and on leaving left instructions for the extraction of all her teeth. She was too sick to sit up. I examined her teeth; there were two broken off, the gums very much inflamed, another very much decayed, *a dens sapientiæ*, and the gum inflamed I decided, and removed the three teeth, and in each sinus put some sulphate of morphia, directing her to come into town as soon as she was able and let me fill some others that were decayed. She came in a few days. I filled several teeth, and she returned home. It was only a short time until the physician sent for me to come and extract all of Mrs. ——— teeth, she was suffering constantly with neuralgia, and did not think there was any other remedy for her case. When I arrived, I found her under the influence of morphia again and suffering very much. She said her physician left word for me to take all her teeth out before I left. I examined the teeth carefully and could find no cause for the pain in them, and by percussion they gave no signs of distress. She complained of the left side of her face giving pain all the time. About two years before this, while filling some teeth for her, she asked me not to press on that side of her face, as it gave her pain. I observed the face looked a little flushed, but thought no more of it until the second visit to the country to see her. While making the examination of her teeth, she requested me not to press on that side of her face. I then examined the mouth of the duct of steno, and found it closed and a circle about the size of a nickel of a bluish color. I then, with a small instrument armed with a little cotton, attempted to enter the mouth of the duct, but found it closed up. I then applied some strong camphor water and wiped the surface dry, and forced a small smooth pointed instrument into the mouth of the duct, and moved the obstruction back a little. By further observation, I found it to be some hard substance about half the size of an ordinary grain of wheat. I then armed the point of a small instrument with cotton wool and passed it into the mouth of the duct, and by turning the instrument the fibers of the cotton were wound around this obstruction, and I succeeded in removing it from its position and passing it through the mouth of the duct; there was some pain to the patient. In tracing the duct I found another obstruction about one-half inch above the mouth of the duct. The patient declined my proceeding further with the case that morning. I took camphor water and carbolic acid *aa*, and with some cotton penetrated up the duct to the next obstruction. Requesting her to return next morning and bring her physician. I thought I had discovered her trouble and would not extract her teeth, as I was fully satisfied they were not the cause of the trouble. I advised her during the day to apply a flannel napkin wet in warm water, to which some camphor water had been added. She did so, and early next morning with her physician was in my office. I told him why I did not extract the teeth, and thought I had discovered the cause of her trouble. After a minute examination, I proceeded to explore the duct, and soon found the way was not clear, and tracing the line of the duct found still another obstruction, and told him of the removal of the one the day before. I told him I proposed the removal of the other already discovered. The physician remarked, "Well, sir, your theory of the case is good; I know nothing of dentistry, and will turn the case over to you. I hope you may be successful; I have had trouble with this case for five or six years, and had arrived at the conclusion that nothing but extracting all her teeth would bring her relief." I examined the eye and found that it was in sympathy all the time. I succeeded in dislodging two more pieces of calculus that had formed in the duct, making three in all. These entirely closed up the passage into the mouth, and prevented the discharge of the saliva. On the removal of these obstructions the saliva spouted beyond the mouth. I passed a small flexible wire, armed with cotton wet in camphor water and carbolic acid, up the duct three or four inches. I directed that she apply the napkin as before to her face, and apply warm water to the mouth of the duct. I had her return for several days to the office, and each visit showed decided improvement. Soon all pain was gone, the eye was relieved, the pain in the face had departed. She left soon, and spent some time at Hugh's Springs for her health; and on her return I was gratified to see her so much improved and hear her say my teeth do not trouble me now, my eye is all right, and the pain and tenderness in my face is all gone. Two years after this she was free from pain and her general health good. The deposits I removed were rough on the surface and looked like the substance of salivary calculus. I intended sending them to the Ohio Dental College, but I lost them. This simple statement of the case may aid some young practitioner, and direct some of our leading men to give the subject thought, and make suggestions of a valuable character to the profession.

---

DEATH PRODUCED BY LIVING FISH.—The *Journal de Medecine d'Algerie* reports eleven cases in which the custom indulged in by fishermen of killing fish with their teeth has been productive of various accidents. In two cases mentioned, a small fish became lodged in the respiratory tract, causing the death of the man.

